# Advances in Tumor Antigen Vaccines: A New Frontier in Cancer Immunotherapy

**DOI:** 10.7150/ijms.120868

**Published:** 2025-10-24

**Authors:** Wanqi Feng, Yifan Zhao, Dongran Yu, Wenyu Jia, Hui Cao, Yuling Zhang, Jie Cao, Zequn Li

**Affiliations:** 1 Department of Gastrointestinal Surgery, The Affiliated Hospital of Qingdao University, Qingdao, 266000, China.; 2 Gastrointestinal Tumor Translational Medicine Research Institute of Qingdao University, Qingdao, 266000, China.; 3 Qingdao Medical College, Qingdao University, Qingdao, 266000, China.; 4 Department of Pharmaceutics, School of Pharmacy, Qingdao University, Qingdao, 266071, China.; 5 Department of Endocrinology, Qingdao Municipal Hospital, Qingdao, 266071, China.

**Keywords:** Tumor antigen vaccines, Tumor-associated antigens, Tumor-specific antigens, Vaccine platforms, Combination therapy.

## Abstract

With the increasing prominence of cancer immunotherapy, therapeutic tumor vaccines have emerged as a promising strategy to enhance antitumor immunity by increasing tumor immunogenicity and activating the patient's immune system to inhibit tumor growth. However, their clinical efficacy is often limited due to insufficient immune cell infiltration, low antigen immunogenicity, and tumor immune escape mechanisms. To address these challenges, various innovative approaches have been explored, including the optimization of tumor antigen selection, the development of advanced vaccine platforms, and the combination of vaccines with other treatment strategies such as radiotherapy, chemotherapy, immune checkpoint inhibitors (ICIs), cytokine therapy, and adoptive T-cell transfer. This review provides a comprehensive summary of the mechanisms underlying tumor antigen vaccines, discusses recent advancements in vaccine design and combinatorial strategies, and assesses their potential to enhance therapeutic outcomes. We also highlight the ongoing challenges and future directions, underscoring the importance of interdisciplinary efforts to realize the full potential of tumor vaccines as a foundation of personalized cancer immunotherapy.

## Introduction

**Figure 1:**
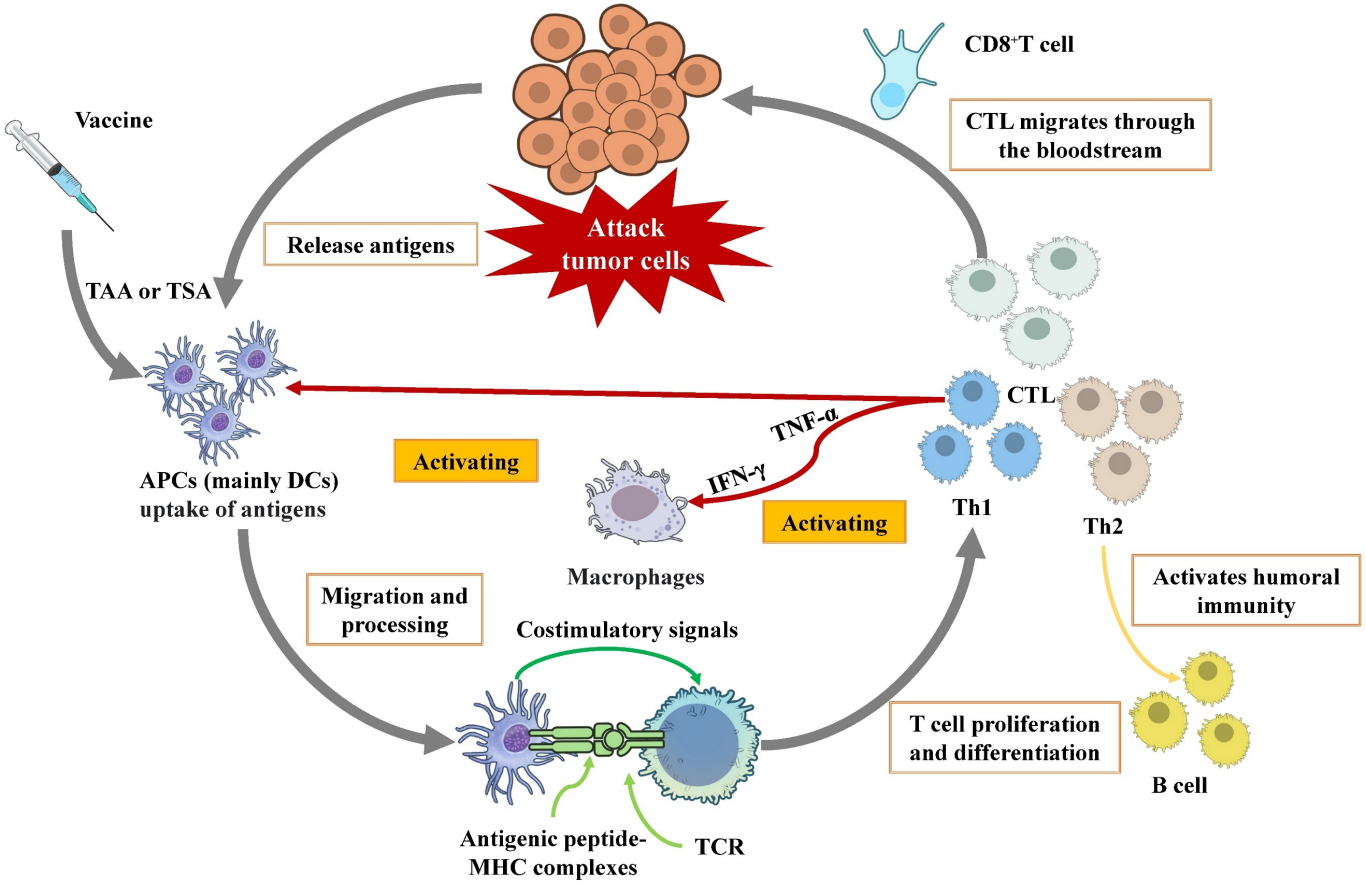
Schematic diagram of the mechanism of therapeutic tumor vaccines

Cancer remains a major global health challenge, with conventional therapies like surgery, chemotherapy, and radiotherapy often yielding limited efficacy in advanced stages due to systemic toxicity and the development of resistance 1. Immunotherapy has revolutionized oncology by harnessing the body's immune system to combat cancer. Among these strategies, ICIs and adoptive cell therapy (ACT) have achieved remarkable clinical success. ICIs function by blocking inhibitory pathways to “release the brakes” on pre-existing tumor-specific T cells, while ACT involves the *ex vivo* expansion and reinfusion of autologous immune cells to directly mediate tumor killing. As shown in Fig. 1, tumor vaccines deliver tumor antigens (proteins, peptides, mRNA, *etc.*) to the immune system, activate initial T cells, and trigger a long-term immune response against specific antigens, which subsequently leading to an anti-tumor effect 2, 3.

Tumor antigens, broadly categorized as tumor-associated antigens (TAAs) and tumor-specific antigens (TSAs), constitute the basis for vaccine design 4. Following administration, vaccine antigens are primarily captured by antigen-presenting cells (APCs), with dendritic cells (DCs) being the most crucial type 5. DCs internalize and process these antigens into short peptides, which are subsequently loaded onto major histocompatibility complex (MHC) molecules. The peptide-MHC complexes are presented on the DC surface 6. T cell activation requires specific recognition of these complexes by the T-cell receptor (TCR), coupled with essential costimulatory signals. Once activated, T cells proliferate and differentiate into distinct functional subsets, primarily cytotoxic T lymphocytes (CTLs) and helper T cell 7. CTL has the capacity to recognize and directly kill tumor cells that express corresponding antigens. This process can occur in three ways: (1) the secretion of pro-inflammatory cytokines, (2) the interaction between Fas ligands and Fas receptors, and (3) the secretion of perforin-containing cytolytic granules 8. Th cells differentiate into Th1 and Th2 subsets. Th1 cells, characterized by IFN-γ and TNF-α secretion, are pivotal for activating and sustaining CTLs, enhancing APC function, and inducing specific antibodies that promote phagocytosis of infected or tumor cells. Th2 cells primarily support humoral immune responses 9.

However, tumors have developed various mechanisms, such as antigen modulation, abnormal expression of immune checkpoint molecules, immunosuppressive cell aggregation, cytokine environment modification, and tumor cell heterogeneity, which pose significant challenges to the efficacy of tumor antigen vaccines 10.

To overcome these challenges, considerable efforts are focused on optimizing antigen selection, developing novel vaccine platforms, and combining vaccines with other modalities. This review provides a comprehensive overview of the recent advances in tumor antigen vaccines, discussing the complexities of antigen selection, platform technologies, and combinatorial strategies that show promise for the future of cancer immunotherapy.

## Tumor Antigens: Targets for Immunotherapy

During carcinogenesis, genetic alterations in normal tissues can lead to the aberrant expression of specific proteins. These tumor-associated antigens are differentially expressed between tumor and normal cells and are recognized by the immune system as foreign, thereby eliciting an immune response against cancer cells 11. Such antigens serve as key targets for developing therapeutic cancer vaccines. However, most tumor antigens are endogenous in origin, which renders them less immunogenic compared to conventional exogenous antigens. Additionally, the heterogeneity of tumor antigens further challenges the identification of optimal targets for vaccination 12, 13. As is shown in Table 1, tumor antigens are broadly classified into two categories: TAAs and TSAs.

### TAAs: Characteristics and Clinical Challenges

Overexpressed antigens are self-proteins present at substantially elevated levels in tumor cells compared to normal tissues. Representative examples encompass carcinoembryonic antigen (CEA), prostate-specific antigen (PSA), melanoma antigen recognized by T cells 1 (MART-1), and gp100 14-16. New York esophageal squamous cell carcinoma 1 (NY-ESO-1) and melanoma-associated antigen family A (MAGE-A), exhibit expression largely confined to immune privileged sites (*e.g.*, testes and ovaries), thereby rendering them attractive targets for vaccine design 17, 18. Oncofetal antigens, normally expressed during fetal development and silenced in adults, can be re-expressed in certain cancers. Targeting strategies leveraging such antigens are exemplified by chimeric antigen receptor (CAR) T-cell therapy 19.

Nevertheless, immunotherapy targeting these distinctive TAAs faces several challenges. Clinical trials evaluating various cancer vaccines based on MAGE-A and NY-ESO-1 antigens across multiple malignancies (e.g., lung, bladder, and skin cancers) indicate that further investigation is necessary to establish their optimal clinical safety, efficacy, and tolerability 20, 21. These findings highlight the necessity of more clearly elucidating the expression mechanisms of TAAs in both normal and malignant contexts. Moreover, additional clinical studies are essential to refine immunotherapeutic strategies and improve outcomes.

To enhance immunogenicity while mitigating autoimmune toxicity, attention is increasingly directed toward TAAs resulting from post-translational modifications (PTMs) or alternative splicing 22-24. PTMs involve chemical alterations of amino acid residues, whereas alternative splicing generates unique protein isoforms through variant exon inclusion. Promising TAAs originating from these mechanisms include CD44v6, STn, and O-GD2 25.

### From TSAs to Personalized Vaccines

TSAs, also known as neoantigens, represent a distinct and highly advantageous class. A defining characteristic of TSAs is their exclusive expression in cancer cells and absence in normal tissues. This tumor-restricted expression classifies them as exogenous antigens, presenting high immunogenicity and capacity to stimulate CD4^+^ and CD8^+^ T cells, thereby eliciting potent immune responses. Crucially, TSAs do not typically induce autoimmune reactions-a key advantage over TAAs. Moreover, they evade central and peripheral immune tolerance mechanisms. Furthermore, central and peripheral immune tolerance does not affect TSAs. As a result, TSA-targeting vaccines hold strong potential for improved efficacy and safety profiles 26.

Emerging clinical evidence supports the promise of TSA vaccines. The TSA vaccine based on uridine mRNA-lipoplex nanoparticles in combination with atezolizumab and chemotherapy induced TSA-specific immune responses that significantly delayed the recurrence of pancreatic cancer. Of note, the personalized mRNA TSA vaccine could be completed within nine weeks, suggesting that mRNA cancer vaccines can be integrated into post-surgery clinical care 27. Given the high heterogeneity of tumor cells, many cancers have been treated with tumor vaccines that target TSAs individually. For example, the synthetic long peptide vaccine for TSAs (#NCT03121677) for follicular lymphoma, the TSAs pulsed DC vaccine (#NCT02956551) for metastatic lung cancer 28, 29. The employment of rapid and effective bioassays facilitates the selection of TSAs with high immunogenicity for each patient.

However, the development of personalized TSA vaccines remains costly and time-intensive. Advances in biomonitoring technologies, novel algorithms, and machine learning are urgently needed to accelerate mutation identification and enhance the screening of T-cell-recognizable epitopes. Beyond personalized approaches, the identification of “public” TSAs offers a promising path toward developing broadly effective, off-the-shelf TSA vaccines. Public TSAs often arise in essential driver genes regulating tumor growth, making them potent therapeutic targets 30, 31. Leveraging pan-cancer proteo-genomics, refined MHC binding prediction, single-cell transcriptomics, and TCR sequencing, researchers are continuously expanding the repertoire of public TSAs. Examples include mutant KRAS peptides, altered driver gene products and RPL22 variants resulting from RNA splicing aberrations across diverse cancers 32-34.

## Platforms for Cancer Vaccine Development

**Figure 2:**
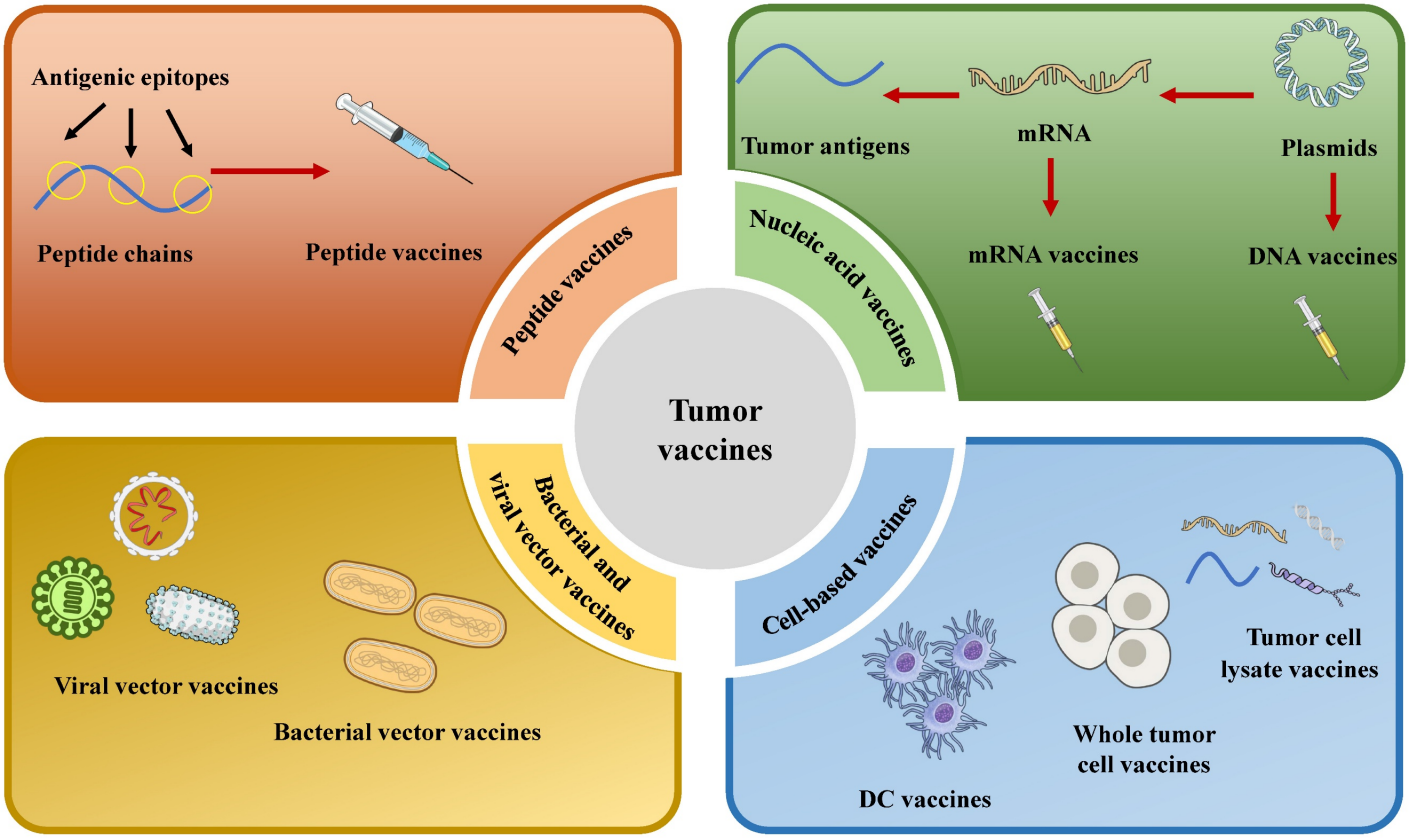
Schematic diagram of the classification of therapeutic tumor vaccines

Selecting an optimal vaccine platform requires careful consideration of multiple factors. Moreover, it is essential to integrate a variety of tumor antigens with multiple vaccine platforms. Vaccine platforms are broadly classified into four types based on their fundamental design: cell-based cancer vaccines, peptide vaccines, viral and bacterial vector vaccines, and nucleic acid vaccines (Fig. 2).

### Peptide Vaccines: Precision and Limitations

Peptide vaccines typically consist of short sequences of approximately 25 amino acids. Shorter peptides exhibit excessively brief half-lives and are highly soluble in serum, which limits their efficacy. A primary objective of these vaccines is to activate CTLs, particularly CD8+ T cells, due to the crucial role of T-cell immunity in cancer immunotherapy. This activation primarily occurs through cross-presentation by APCs, a process crucial for mediating antitumor responses 35, 36.

Peptide vaccines offer several advantages, such as high specificity, a reduced risk of autoimmune reactions, and high safety. For example, the amphiphilic vaccine Amph-CpG-7909 (targeting mKRAS with a CpG adjuvant) was well-tolerated and induced mKRAS-specific T-cell responses in 84% of patients (21/25) 37. Another first-in-human study of the peptide vaccine TAS0313 patients with advanced solid tumors confirmed its safety, tolerability, and ability to induce immune response 38. Moreover, the direct binding of defined peptide epitopes to MHC molecules makes peptide vaccines valuable tools for identifying novel tumor-specific T cell epitopes, enabling efficient *in vitro* screening using APCs and T cells or peptide-MHC complex analyses.

However, peptide vaccines also face significant limitations, including complex manufacturing processes, high production costs, and a tendency to elicit weak immune responses that are often insufficient within the immunosuppressive tumor microenvironment 39, 40. To address these limitations, self-assembling peptide vaccines have emerged as a promising advanced platform. By leveraging non-covalent interactions to form well-ordered nanostructures (e.g., nanofibers, nanoparticles, hydrogels), self-assembling peptides can present high densities of antigens in a multivalent manner, enhancing uptake by APCs and potently activating both cellular and humoral immunity without the need for additional adjuvants. This platform integrates the specificity of peptide vaccines with improved immunogenicity and stability, presenting a robust approach to cancer immunotherapy 41.

### Nucleic Acid Vaccines: A Modern Modality

Nucleic acid vaccines, comprising both DNA and RNA platforms, function by delivering genetic material that encodes tumor antigens into host cells to elicit an immune response. The global response to the COVID-19 pandemic has accelerated significant advancements in nucleic acid vaccine technology, exemplified by the widespread clinical use of mRNA vaccines against SARS-CoV-2. This milestone highlights the translational potential of nucleic acid vaccines. Importantly, similar to other vaccine modalities, they demonstrate considerable promise for integration into cancer immunotherapy strategies 42.

#### DNA Vaccines: Challenges in Sustained Expression and Delivery

A key advantage of DNA vaccines is their capacity to enable sustained expression of tumor antigens, thereby inducing more prolonged immunogenicity compared to peptide platforms. Advancements in plasmid engineering have significantly enhanced this technology, with contemporary constructs incorporating strong promoters and immunostimulatory CpG motifs to enable the efficient and cost-effective expression of multiple antigens 43, 44.

Despite these advantages, the clinical application of DNA vaccines faces several limitations. A significant challenge is their suboptimal transfection efficiency, which is partly attributable to variations in the composition of cellular and nuclear membranes across different cell types. Plasmids must enter the cytoplasm via pinocytosis or endocytosis without being degraded-a process that remains inefficient. These delivery challenges contribute to issues such as low immunogenicity, potential autoimmune reactions, and the risk of host genomic integration 45. Physical delivery methods, including electroporation, gene guns, and sonoporation, are commonly employed to enhance plasmid uptake; however, their efficacy remains limited 46-48. To address these challenges, researchers are developing non-viral nanoparticle-based delivery systems utilizing lipid nanoparticles and cationic polymers 49.

Furthermore, certain adjuvants have proven effective for enhancing DNA vaccine efficacy, particularly nano-agonists targeting the stimulator of interferon genes (STING) pathway. The recognition of cytosolic DNA is mediated through the STING signaling axis, which induces type I interferon (IFN) production and contributes to the self-adjuvating properties of DNA plasmids. In preclinical studies, STING agonists, such as those based on manganese-doped silica nanoparticles, have been shown to enhance immune responses induced by plasmid DNA vaccines. These agents also facilitate DC activation and migration, thereby enhancing anti-tumor immunity 50.

#### RNA Vaccines: Rapid Development and Clinical Translation

Four primary classes of RNA have been employed in vaccine development: messenger RNA (mRNA), circular RNA (circRNA), trans-amplifying RNA (taRNA), and virus-derived self-amplifying RNA (saRNA) 51. Among these, saRNA is particularly notable due to its ability to replicate within cells, leading to amplified antigen expression compared to conventional non-replicating mRNA. This property results in a prolonged intracellular half-life and lower required doses 52. Conventional mRNA vaccines contain encoded antigen sequences flanked by 5' and 3' untranslated regions but do not self-replicate. mRNA vaccines exhibit a favorable safety profile-being non-infectious, non-integrating, and biodegradable-while also offering advantages in rapid, cost-effective development. These advantages-especially their rapid development timeline and manufacturing scalability-have established mRNA platforms as leading candidates for clinical translation, with numerous vaccine candidates currently progressing through clinical trials (Table 2).

mRNA vaccine technology has advanced rapidly in recent years. The carcinoembryonic antigen Claudin 6 (CLDN6), for instance, is highly expressed in a variety of solid tumors 53. One study evaluated the efficacy of CAR T cells targeting CLDN6 in combination with an amplifying RNA vaccine (CARVac). The CARVac vaccine consists of a nucleoside-modified mRNA encoding CLDN6, packaged into lipid nanoparticles (LNPs) to enhance delivery efficiency and stability 54. These LNPs protect mRNA from enzymatic degradation and facilitate its uptake by APCs. It has been shown that complexing mRNA with positively charged liposomes forms RNA-LPX, which shields the RNA from nucleases and promotes efficient entry into APCs. Moreover, LNPs enhance vaccine immunogenicity by improving antigen uptake and presentation. Clinical trials have corroborated these findings, indicating favorable safety and immunogenicity profiles. The BNT211-01 trial demonstrated that although the combination of CLDN6 CAR-T and CARVac was safe, the assessment of efficacy was limited by the small sample size, high patient heterogeneity, and the absence of control groups, thereby complicating the isolation of the vaccine's contribution. The delayed vaccination likely missed the optimal window for immune stimulation and was unable to compensate for lymphodepletion or counteract the immunosuppressive tumor microenvironment. The absence of predictive biomarkers further limits clinical applicability. Future trials should optimize dosing schedules, increase cohort sizes, and investigate the sequencing of combination therapies 55.

Despite these advances, mRNA vaccines face several challenges, including limited stability, intricate regulatory pathways, suboptimal immunogenicity, inefficient delivery, and difficulties in production and storage. To overcome these limitations, researchers are pursuing strategies such as optimizing mRNA sequence and structure, refining delivery systems, and elucidating underlying immune mechanisms. Efforts are also underway to improve manufacturing processes and storage conditions to enable safe and effective vaccine deployment 56, 57.

circRNAs represent a distinct class of single-stranded RNAs with covalently closed circular structures. This conformation confers resistance to exonuclease degradation and enables sustained protein expression *in vivo*. Preclinical studies of circRNA encapsulated in LNPs have demonstrated potent antitumor effects in mouse models, owing to improved cytosolic delivery and reduced innate immunogenicity compared to linear mRNA 58, 59.

### Cell-Based Vaccines: Harnessing Cellular Immunity

Cell-based cancer vaccines represent a class of immunotherapies that utilize whole cells-either as carriers or as central immunogenic components-to elicit anti-tumor immune responses. This category primarily includes tumor cell vaccines and DC vaccines.

Tumor cell vaccines are prepared from autologous or allogeneic tumor cells. Whole-cell vaccines employ tumor cells that have been inactivated through physical, chemical, or genetic methods to eliminate tumorigenicity while preserving immunogenicity. Alternatively, tumor cell lysate vaccines utilize solubilized tumor materials containing a broad spectrum of TAAs, which may help overcome antigenic heterogeneity and immune escape 60-62.

DC vaccines leverage the potent antigen-presenting capacity of DC to stimulate anti-tumor immunity. These vaccines are produced by loading TAAs or tumor-specific antigens onto DCs *ex vivo*, which are then reinfused to activate T-cell responses against tumor cells. In 2010, the U.S. FDA approved Sipuleucel-T, the first therapeutic DC vaccine for metastatic prostate cancer, marking a milestone in cell-based immunotherapy 63. Nonetheless, its clinical application remains limited, and no other DC vaccine has subsequently obtained FDA approval.

Nevertheless, DC vaccines have exhibited promising safety and efficacy in numerous studies, thereby supporting their assessment across an expanding spectrum of cancers. For example, a single-arm, dual-center pilot study (ChiCTR-ONC-16009100, NCT02956551) involving advanced lung cancer reported favorable outcomes following the use of a personalized TSA-pulsed autologous DC vaccine (Neo-DCVac) 29. DC vaccines continue to face significant challenges in achieving consistent clinical efficacy. Other clinical trials have explored DC vaccines targeting single antigens such as WT1, HER2, and IL-13Rα2 in glioma, with some showing preliminary efficacy. To address tumor heterogeneity, multi-antigen DC vaccines such as ICT-107 have been developed and have demonstrated promising results in early-phase clinical trials 64, 65. Moreover, in cancers such as colorectal carcinoma, the majority of patients have not experienced substantial survival benefits from DC vaccines, highlighting the necessity for enhanced strategies in vaccine design, delivery, and combination therapies to overcome immunosuppressive tumor microenvironments 66.

A significant technical challenge in the production of DC vaccines is the limited availability of peripheral blood DCs. Second-generation DC vaccines often use monocyte-derived DCs (Mo-DCs) as a more feasible source of APCs. However, Mo-DCs exhibit significant functional and genetic differences compared to natural DCs, including impaired cross-presentation, limited migratory capacity, and poor survival after infusion, resulting in inefficient lymph node homing and T-cell priming. The limited availability and low purity of other DC subsets, coupled with technical challenges in their isolation and culture, further constrain broader application 67, 68. In addition, the researchers have developed a novel whole tumor cell vaccine platform, which successfully solves the two core bottlenecks of traditional whole tumor cell vaccines through intracellular gelation technology combined with cell surface engineering of CD47 blockade and damage-related molecular pattern exposure 69.

### Viral and Bacterial Vectors: Engineered Delivery Platforms

Viral and bacterial vector vaccines employ engineered viruses or bacteria to deliver genes encoding pathogen antigens into host cells. Through infection, these vectors facilitate the expression of target antigens, thereby eliciting specific immune responses. A key advantage of such vaccines is their high immunogenicity, enabling efficient cellular entry and the induction of durable immunity.

Viruses attenuated to eliminate pathogenicity while maintaining infectivity enable highly efficient gene delivery. Among these, oncolytic viruses (OVs), which selectively replicate in cancer cells, have demonstrated considerable clinical potential. For instance, vaccinia virus (VV), a member of the Poxviridae family, has been utilized both as a vaccine vector and an oncolytic agent. Preclinical and early-phase clinical studies indicate that VV-based vaccines, particularly in combination with standard treatments such as ICIs, exhibit notable efficacy in advanced non-small cell lung cancer and metastatic breast cancer 70, 71. Several OV therapies, including Rigvir and Oncorine, have already obtained regulatory approval 72, 73. Nonetheless, selecting an optimal viral vector involves balancing safety and immunogenicity: vesicular stomatitis virus (VSV) offers high immunogenicity but raises biosafety concerns; parainfluenza virus (PIV) vectors exhibit improved safety profiles but relatively weaker immune stimulation; and adenoviral vectors (AdVs) strike a balance between immunogenicity and sustained antigen presentation 74.

Regarding bacterial vectors, gram-negative bacteria secrete outer membrane vesicles (OMVs), nanoscale bilayer structures enriched with immunostimulatory components such as lipopolysaccharides and proteins. Through genetic engineering, OMVs can be designed to display tumor antigens, forming customizable vaccine platforms. “Plug-and-Display” technology enables rapid antigen conjugation to ClyA proteins on OMV surfaces, facilitating simultaneous DC activation and antigen cross-presentation. In preclinical models of melanoma and colorectal cancer, OMV-based vaccines have been shown to induce robust T-cell responses, suppress tumor growth and metastasis, and establish long-term immunological memory. Although OMV-based approaches date back to early tuberculosis vaccine research in the 20^th^ century, advances in genetic engineering have now unlocked their full therapeutic potential.

## Combination Therapies: Synergistic Strategies

**Figure 3:**
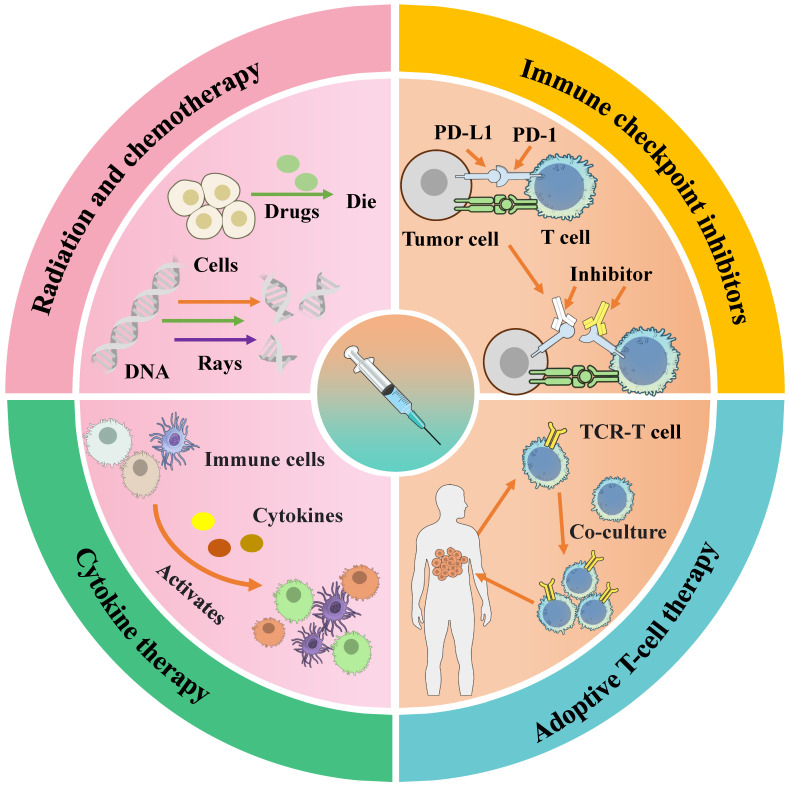
Schematic diagram of therapies used in combination with tumor vaccines

To overcome the inherent limitations of monotherapy, tumor antigen vaccines are increasingly being integrated with established and emerging cancer treatments. These combinations aim to remodel the tumor microenvironment and amplify antigen-specific immunity, as summarized in Fig. 3. The combination of tumor antigen vaccines with other modalities aims to overcome specific barriers in the cancer immunity cycle. The advantages, and limitations of these strategies are summarized in Table 3.

### Radiation and Chemotherapy: Priming the Immune Landscape

Radiation therapy (RT) is a widely used modality for treating malignant tumors through the application of high-energy radiation. Its fundamental mechanism involves inducing DNA damage in tumor cells, thereby inhibiting their proliferation and survival. The primary goals of RT are to eradicate tumor cells and reduce tumor volume 75.

RT not only reduces tumor burden but also enhances the infiltration of effector immune cells into the tumor microenvironment, thereby addressing a key limitation of immunotherapy. Consequently, combining RT with vaccines represents a promising strategy to amplify anticancer immunity 76. For instance, the novel adeno-associated virus (AAV)-based vaccine “meAAV”, when administered alongside RT, improves antigen presentation and sustains TSA-specific CTL responses. This combination therapy significantly enhances CTL activity, promotes their infiltration into tumors, and alleviates local immunosuppression 77. In a 4T1 breast cancer mouse model, the combination of stereotactic ablative radiotherapy and a cancer vaccine targeting fibroblast-activating protein-alpha (FAP-α) effectively suppressed metastatic growth 78.

Chemotherapy, a fundamental component of cancer treatment, employs cytotoxic agents to systematically target and eliminate rapidly dividing tumor cells 79. However, its efficacy is often compromised by tumor heterogeneity and the emergence of drug resistance. Growing evidence supports the combination of chemotherapy with immunotherapy to improve antitumor responses 80, 81. For instance, the personalized TSA vaccine NEO-PV-01, administered in combination with chemotherapy and the anti-PD-1 antibody pembrolizumab as a first-line treatment for metastatic non-squamous non-small cell lung cancer, was well tolerated and elicited TSA-specific CD4+ T cell responses 82. A phase II study involving patients with metastatic androgen-independent prostate cancer demonstrated that the combination of docetaxel (DTX) and a vaccine-based immunotherapy regimen was safe, did not compromise vaccine-induced T cell responses, and resulted in improved clinical outcomes 80.

The integration of chemoradiation with tumor antigen vaccines has been applied in clinical settings, demonstrating enhanced treatment efficacy. This multimodal approach helps overcome the lack of specificity associated with conventional chemoradiation and chemotherapy, thereby advancing the field of precision medicine.

### ICIs: Releasing the Brakes on Immunity

Combining tumor antigen vaccines with ICIs represents a rational strategy to counteract tumor-induced T-cell exhaustion. As shown in Table 3, vaccines prime and expand antigen-specific T cells, ICIs remove inhibitory signals, enabling robust and durable cytotoxic responses. These inhibitory signals encompass not only the classical PD-1/PD-L1 pathway but also innate immune checkpoints mediated by macrophages, such as the CD47-SIRPα axis and the emerging CD24-Siglec10 axis. CD24, highly expressed on various tumor cells, binds to Siglec-10 on macrophages, transmitting a “don't eat me” signal that inhibits phagocytosis and facilitates tumor immune escape. Targeting this axis has become a new hotspot in cancer immunotherapy 2. By interfering with inhibitory signals from tumor cells to immune cells, ICIs facilitate the targeting and destruction of tumors by activated T cells 83. The combination of tumor antigen vaccines with ICIs has been shown to improve the immune system's ability to recognize and eliminate cancer cells.

Preclinical studies have demonstrated a potent synergistic effect between tumor antigen vaccines and ICIs, leading to substantial suppression of tumor growth. For example, in hepatocellular carcinoma (HCC) models, the combination of an alpha-fetoprotein (AFP) vaccine and anti-PD-L1 therapy led to significant inhibition of tumor progression in the majority of liver lesions, indicating that vaccine-primed T-cell responses can be effectively activated by checkpoint blockade 84. In clinical settings, a phase II trial involving colorectal cancer patients revealed that the GVAX colon vaccine combined with cyclophosphamide and pembrolizumab induced biochemical responses (≥30% reduction in CEA levels) in 41% (7/17) of patients with mismatch repair-proficient tumors 85. Although the trial did not meet its primary endpoint, the considerable biochemical response rate highlights the potential of combinatorial immunotherapy even in immunologically “cold” tumors, supporting further investigation of vaccine-ICI strategies beyond hypermutated cancers.

Beyond HCC and colorectal cancer, this combinatorial strategy has shown efficacy in various other malignancies. In metastatic castration-resistant prostate cancer (mCRPC), the integration of ICIs with androgen deprivation therapy (ADT) and tumor vaccines has exhibited promise not only for prostate cancer but also for melanoma and lung cancer 86. Second-generation ICIs combined with Treg depletion strategies and murine cancer vaccines synergized in a CD8+ T cell-dependent manner, reducing tumor growth and improving survival 87. In Mlh1-deficient mice with gastrointestinal tumors, combining a tumor vaccine with anti-PD-L1 therapy significantly extended survival, increased T cell infiltration, and reduced macrophages, neutrophils, and myeloid-derived suppressor cells (MDSCs) 88.

Overall, the combination of tumor antigen vaccines with ICIs holds considerable potential for enhancing treatment outcomes and advancing personalized and precision cancer medicine.

### Cytokine Therapy: Amplifying Immune Signals

Cytokines are a diverse class of small, soluble polypeptides or glycoproteins that stimulate and regulate the activation and proliferation of immune cells. Although cytokines can enhance immune responses, they do not directly target tumors and rely on the host's pre-existing immune activity. Consequently, although cytokines demonstrate limited efficacy as monotherapies, they can substantially enhance antitumor immunity when used in conjunction with tumor vaccines 89.

Interleukin-15 (IL-15) has encountered obstacles in clinical application, including systemic toxicity, insufficient immune stimulation, and a short half-life, issues that are largely attributable to its widespread receptor distribution and rapid clearance. To overcome these challenges, a biomimetic nano-vaccine termed biNV-IL-15 has been developed. This platform consists of genetically engineered DC membrane vesicles that enable targeted delivery of both IL-15 and antigen/MHC complexes to antigen-specific CTLs, facilitating multivalent IL-15 self-presentation. Preclinical studies indicate that biNV-IL-15 effectively activates CD8+ T cells *in vitro*, extends systemic circulation, enhances accumulation in lymphoid organs, and promotes durable immune memory-all while exhibiting a favorable safety profile 90.

In summary, combining cytokine therapy (particularly IL-15) with cancer vaccines enhances antigen-specific immunity and reduces systemic toxicity. Furthermore, the integration of IL-15 with ICIs may also improve immunotherapeutic outcomes. Innovative delivery platforms, such as the biNV-IL-15 nano-vaccine, demonstrate that cytokine delivery can be precisely targeted to enhance efficacy and minimize systemic toxicity, paving the way for their clinical translation in combination regimens.

## Conclusion

Despite significant progress in tumor antigen vaccines, key challenges persist. These include high research and manufacturing costs, limited access to personalized vaccines, tumor immune evasion mechanisms, and potential autoimmune reactions. Addressing these issues requires interdisciplinary collaboration among immunology, oncology, genetics, and engineering. Further clinical studies are also essential to improve long-term safety and efficacy.

In summary, therapeutic tumor antigen vaccines represent a major advance in cancer immunotherapy, offering a targeted and individualized treatment approach. By utilizing the immune system to eliminate tumor cells, they hold transformative potential for improving patient outcomes. Continued innovation in technology and deeper understanding of tumor biology and immunology are expected to drive future progress, potentially establishing these vaccines as a standard cancer treatment option.

## Figures and Tables

**Table 1:** The characteristics of TSAs and TAAs

	TSAs	TAAs
Expression	Expressed in tumor cells while normal cells do not express Can be produced by carcinogenic viruses	Expressed in both tumor cells and some normal cells Usually highly expressed in tumor cells
Origination	Novel polypeptide chains with mutations primarily driven by cancer	Mainly from gene amplification or post-translational modifications
Example	HPV E6/E7 viral neoantigen	Cancer-testis antigens and oncofetal antigens

**Table 2:** Clinical trials on mRNA vaccines

Sponsoring institution	Tumor types	Study phase	Clinical Trials. gov identifier
University of Florida	Recurrent adult glioblastoma	Phase I	NCT06389591
University of Florida	Pediatric recurrent intracranial malignancies and other systemic solid tumors	Phase I	NCT05660408
University of Florida	Pediatric high-grade gliomas and adult glioblastoma	Phase I	NCT04573140
Chinese Academy of Medical Sciences	Advanced solid tumors	Phase I	NCT06610227
Duke University	WHO grade IV malignant glioma	Phase I	NCT05283109
BioNTech Cell & Gene Therapies GmbH	CLDN6-positive relapsed or refractory advanced solid tumors	Phase I	NCT04503278
University of Florida	Medulloblastoma	Phase I	NCT06514898
BioNTech SE	Head and neck cancer	Phase II	NCT04534205

**Table 3:** Comparison of combination strategies with tumor antigen vaccines

Combination strategy	Key advantages	Limitations and challenges
Radiotherapy	Create an* in situ* vaccine effect; Reverse “cold” tumors to “hot” tumors; Provide localized and focused immune activation.	Abscopal effect is rare; Potential for systemic immunosuppression with high doses; Toxicity to healthy tissues.
Chemotherapy	Employ lymphodepletion to enhance homeostatic cytokine-driven T-cell expansion; Synergize with various vaccine platforms.	Cytotoxicity can deplete immune effectors; Triggers systemic toxicity; Careful timing required.
ICIs	Address a key mechanism of vaccine resistance; Generate durable, memory responses; “Rescue” dysfunctional T cells.	Risk of irAEs; High cost; Requires pre-existing T-cell infiltration.
Cytokine therapy	Directly amplify the immune response initiated by the vaccine; Enhance NK cell cytotoxicity.	Severe systemic toxicity; Short half-life; Pleiotropic effects.

ICIs: Immune checkpoint inhibitors; irAEs: immune-related adverse events.

## Abbreviations

ICIs: immune checkpoint inhibitors
ACT: adoptive cell therapy
CAR-T cells: chimeric antigen receptor T cells
TAAs: tumor-associated antigens
TSAs: tumor-specific antigens
APCs: antigen-presenting cells
DCs: dendritic cells
MHC: major histocompatibility complex
TCR: T-cell receptor
CTLs: cytotoxic T lymphocytes
Th1: T helper 1 cells
IFN-γ: interferon-γ
TNF-α: tumor necrosis factor-α
CEA: carcinoembryonic antigen
PSA: prostate-specific antigen
MART-1: melanoma antigen recognized by T cells 1
NY-ESO-1: New York esophageal squamous cell carcinoma 1
MAGE-A: melanoma-associated antigen family A
PTMs: post-translational modifications
KRAS: Kirsten rat sarcoma viral oncogene homolog
RPL22: ribosomal protein L22
COVID-19: coronavirus disease-2019
SARS-CoV-2: severe acute respiratory syndrome coronavirus 2
CpG motifs: cytosine-phosphate-guanine motifs
STING: stimulator of interferon genes
mRNA: messenger RNA
circRNA: circular RNA
saRNA: self-amplifying RNA
CLDN6: claudin 6
LNPs: lipid nanoparticles
FDA: food and drug administration
HER2: human epidermal growth factor receptor 2
IL-13Rα2: interleukin-13 receptor alpha 2
Mo-DCs: monocyte-derived dendritic cells
Ovs: oncolytic viruses
VV: vaccinia virus
VSV: vesicular stomatitis virus
PIV: parainfluenza virus
AdVs: adenoviral vectors
OMVs: outer membrane vesicles
RT: radiation therapy
AAV: adeno-associated virus
FAP-α: fibroblast-activating protein-α
DTX: docetaxel
PD-1: programmed death protein 1
PD-L1: programmed death-ligand 1
HCC: hepatocellular carcinoma
AFP: α-fetoprotein
mCRPC: metastatic castration-resistant prostate cancer
ADT: androgen deprivation therapy
MDSCs: myeloid-derived suppressor cells
IL-15: interleukin-15
